# Specific Stacking of Key Molecules to Enhance Skin Care Outcomes: Synergistic Effects of Heparan Sulfate Analog and Tetrahexyldecyl Ascorbate Combination

**DOI:** 10.1111/jocd.71103

**Published:** 2026-08-21

**Authors:** Vivian Bucay, Rosalyn George, Anna Guiotto, Alessandra Pecorelli, Emmanuel Ike, Angel S. Byrd

**Affiliations:** ^1^ Bucay Center for Dermatology and Aesthetics San Antonio Texas USA; ^2^ Wilmington Dermatology Cosmetic and Research Center Wilmington North Carolina USA; ^3^ Department for Animal Science, Plants for Human Health Institute, NC Research Campus North Carolina State University Kannapolis North Carolina USA; ^4^ Department of Environmental and Prevention Sciences University of Ferrara Ferrara Italy; ^5^ Department of Food, Bioprocessing and Nutrition Sciences, Plants for Human Health Institute North Carolina State University Kannapolis North Carolina USA; ^6^ Howard University College of Medicine Washington DC USA; ^7^ Department of Dermatology Howard University College of Medicine Washington DC USA

## 1 Introduction

The skin serves as the primary cutaneous barrier interfacing the host with the external environment. It protects against physical, chemical, and biological insults while regulating body temperature, preventing transepidermal water loss, and providing sensory input [1]. Consequently, maintaining skin health is paramount, as it constitutes the body's first line of defense against environmental aggressors [2]. However, the cumulative impact of environmental exposures, primarily ultraviolet radiation and pollutants, accelerates extrinsic aging through the generation of reactive oxygen species (ROS) [3] forming advanced glycation end products (AGEs) that cross‐link the dermal matrix, promoting stiffening, discoloration, and loss of integrity [4, 5, 6, 7]. These insults drive ROS‐mediated extracellular matrix breakdown and inflammatory cascades, culminating in photoaging phenotypes and underscoring the need for targeted interventions.

To counteract these multifactorial damage pathways, dermatological formulations have traditionally incorporated antioxidants such as L‐ascorbic acid or tetrahexyldecyl (THD) ascorbate. Although effective against oxidative stress, there are severe drawbacks from instability and lack of penetration, and mitigating with higher concentrations leads to irritation and inflammation in sensitive skin. To maximize therapeutic impact, intentional pairing of ingredients with independent defined purposes is critical. Simply adding more actives to a formulation does not guarantee better outcomes [8].

In this framework, the bioactive molecule (heparan sulfate analog/HSA) with its unique anti‐inflammatory and regenerative mechanisms of action can optimize the efficacy and mitigate the irritating effects of the THD ingredient. This approach targets multifactorial photoaging processes, such as oxidative stress and inflammatory cascades, from multiple independent entry points, resulting in amplified efficacy through nonadditive interactions [4, 9, 10]. To confirm this hypothesis, researchers evaluated the co‐formulation both ex vivo and clinically to substantiate the optimization effects of this intended biological stacking approach.

The present study systematically examined the co‐formulation's ability to synergistically counteract oxidative damage, DNA lesions, glycation, impaired collagen synthesis, and extracellular matrix disruption. It was hypothesized that topical application of HSA with THD ascorbate would outperform individual components by leveraging HSA's platform role to not only manage the cellular environment but also optimize THD ascorbate delivery, function, and tolerability, thereby restoring dermal homeostasis more effectively [11].

The ex vivo assessment utilized triplicate human skin explants that underwent UV exposure followed by treatment with Senté's Defense C Serum. Immunohistochemical analysis quantified 4‐hydroxynonenal (4‐HNE) as a marker of oxidative stress, phosphorylated histone H2AX (gamma‐H2AX) as a marker of DNA damage, and dermal advanced glycation end products (AGEs). Paired‐sample *t*‐tests compared posttreatment scores (UV + Defense C) to UV‐only; statistical significance was set at *p* < 0.05.

A 12 week, dual‐center single‐arm prospective interventional study was then conducted at two independent sites from April 2025 to August 2025. Prior to participation, all study participants signed informed consent/photo release and underwent a 2 week washout period. Eligible participants were female adults aged 25‐69 (Fitzpatrick skin types I‐VI) with perceived dull skin and skin tone unevenness and in good general health. Informed Consent Form (ICF) was obtained from each participant prior to initiating the study describing reasons for the study, possible adverse effects, associated risks and potential benefits. Signature of a Photography Release Forms were signed, indicating the subject's authorization for release of any photographs obtained during the study.

All products, including Senté's Defense C Serum, are available on the market and therefore no IRB was required.

Enrollment required willingness to use only provided homecare products, attend five visits over 14 weeks, apply SPF 30 sunscreen daily, minimize sun exposure, and discontinue anti‐aging products during a 2 week washout period. Key exclusion criteria included pregnancy or breastfeeding; active inflammatory skin conditions; history of immunosuppression or autoimmune disease; cancer treatment within 12 months; laser resurfacing, radiofrequency, or ultrasound skin‐tightening within 12 months; neurotoxin or filler injections within 6 months; or resurfacing procedures within 4 weeks.

Sixteen subjects (Table 1) applied Senté's Defense C Serum twice daily alongside a gentle cleanser, moisturizer, and SPF 30 sunscreen (once daily). Assessments were conducted at screening; baseline (2‐week washout); Week 4; Week 8; and Week 12. Digital photographs (Visia CR Imaging System), as well as validated investigator assessments, were completed, including skin brightening efficacy and tolerability (Table 2). Additionally, subjects completed a Self‐Assessment Questionnaire at Week 4, Week 8, and Week 12.

**TABLE 1:** Participant distribution and demographics.

		*N*
Enrollment	Subjects enrolled	16
	Subjects completed	16
Age	Mean	46
	Minimum	25
	Maximum	69
Sex	Female	16
	Male	0
Race	White (Fitzpatrick skin types I–IV)	16

**TABLE 2:** Investigator's assessments.

Investigator's assessments: photodamage skin efficacy parameters (5‐Point Scale)						
Score	Fine lines	Wrinkles	Skin tone	Skin radiance	Skin texture (overall skin smoothness)	Global photodamage
0 = none	No evidence of fine lines/wrinkles	Skin is completely smooth	Skin is uniform in color/complexion	Skin has youthful glow	Skin is even, smooth, soft/nonrough texture	No evidence
1–2 = mild	Mild fine lines	Mild texture	Mildly uneven	Mildly radiant	Mildly textured	Mild
3–4 = moderate	Moderate fine lines	Moderate texture	Moderately uneven	Moderately radiant	Moderately textured	Moderate
5 = worse possible/severe	Numerous fine lines	Skin is roughly textured	Skin is uneven and blotchy	Skin is dull/lacking glow	skin is rough, even, bumpy or not level texture	Severe/worse possible

Investigator's assessments: skin brightening efficacy parameters (5‐Point Scale)				
Score	Skin clarity	Skin luminosity	Skin brightness	Investigators tolerability assessment
0 = none	Clear skin without black and white heads, pimples, and spots (red or brown age spots)	Skin is radiant and smooth	Skin is bright/has a youthful glow	None
1–2 = mild	Mildly unclear	Mildly dull/rough	Mildly dull/nonuniform	Mild
3–4 = moderate	Moderately unclear	Moderately dull/rough	Moderately dull/nonuniform	Moderate
5 = worse	Worse possible	Skin is dull, rough texture	Skin is dull/nonuniform in color	Severe/worse possible

**FIGURE 1:**
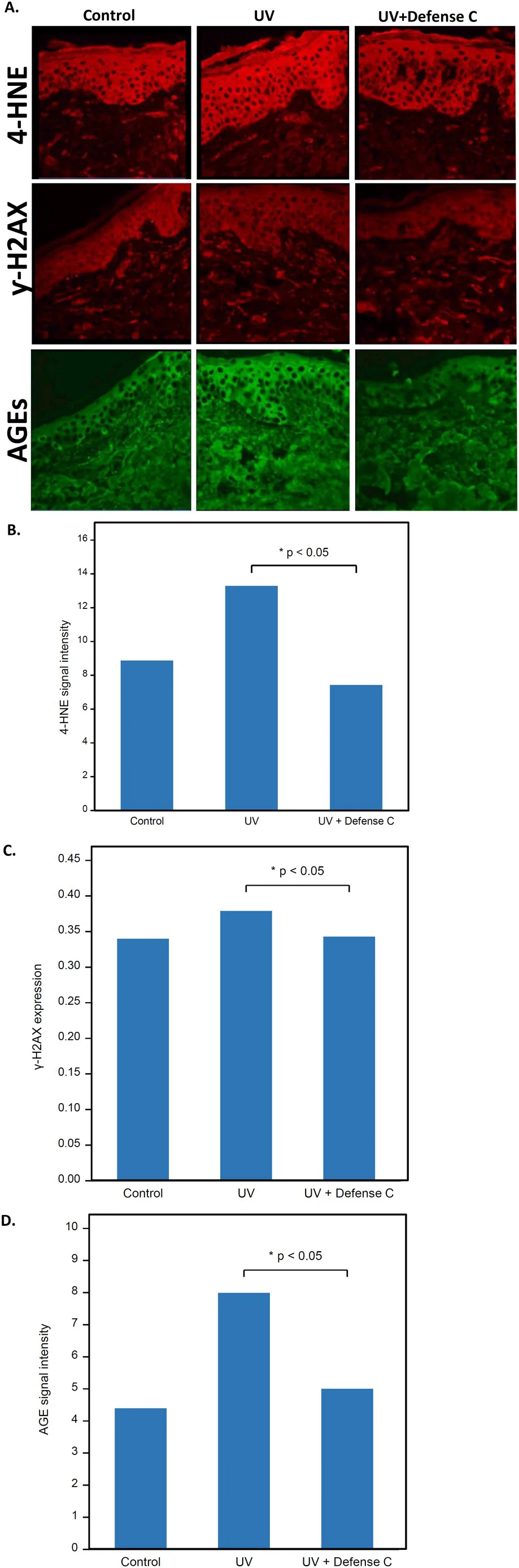
Protection against UV‐induced molecular damage by HSA‐THD (Day 1). Treatment with UV + Defense C reduced oxidative stress, DNA damage, and glycation compared with UV exposure alone. (A) Representative 4‐HNE immunostaining demonstrating lipid peroxidation after UV exposure and reduction with Defense C treatment (*upper panels*), γ‐H2AX immunostaining indicating UV‐induced DNA damage (*middle panels*), dermal advanced glycation end products (AGEs) (*lower panels*). (B) Quantification of 4‐HNE signal intensity. (C) Quantification of γ‐H2AX expression. (D) Quantification of dermal AGEs. *Statistically significant (*p* < 0.05)—UV vs. UV + Defense C; 4‐HNE, 4‐hydroxynonenal; γ‐H2AX, gamma‐histone H2AX; AGEs, advanced glycation end products; HSA‐THD, Heparan Sulfate Analog and Tetrahexyldecyl Ascorbate (Defense C).

**Ex Vivo**: At 24 h, the co‐formulation produced statistically significant reductions relative to UV‐exposed skin (Day 1): 4‐HNE by 44%, gamma‐H2AX by 9.5%, and dermal AGEs by 37% (Figure 1A–D). Protective effects were observed after a single application, consistent with mechanisms of microenvironmental stabilization and antioxidant support. No cytotoxic events occurred.

**FIGURE 2:**
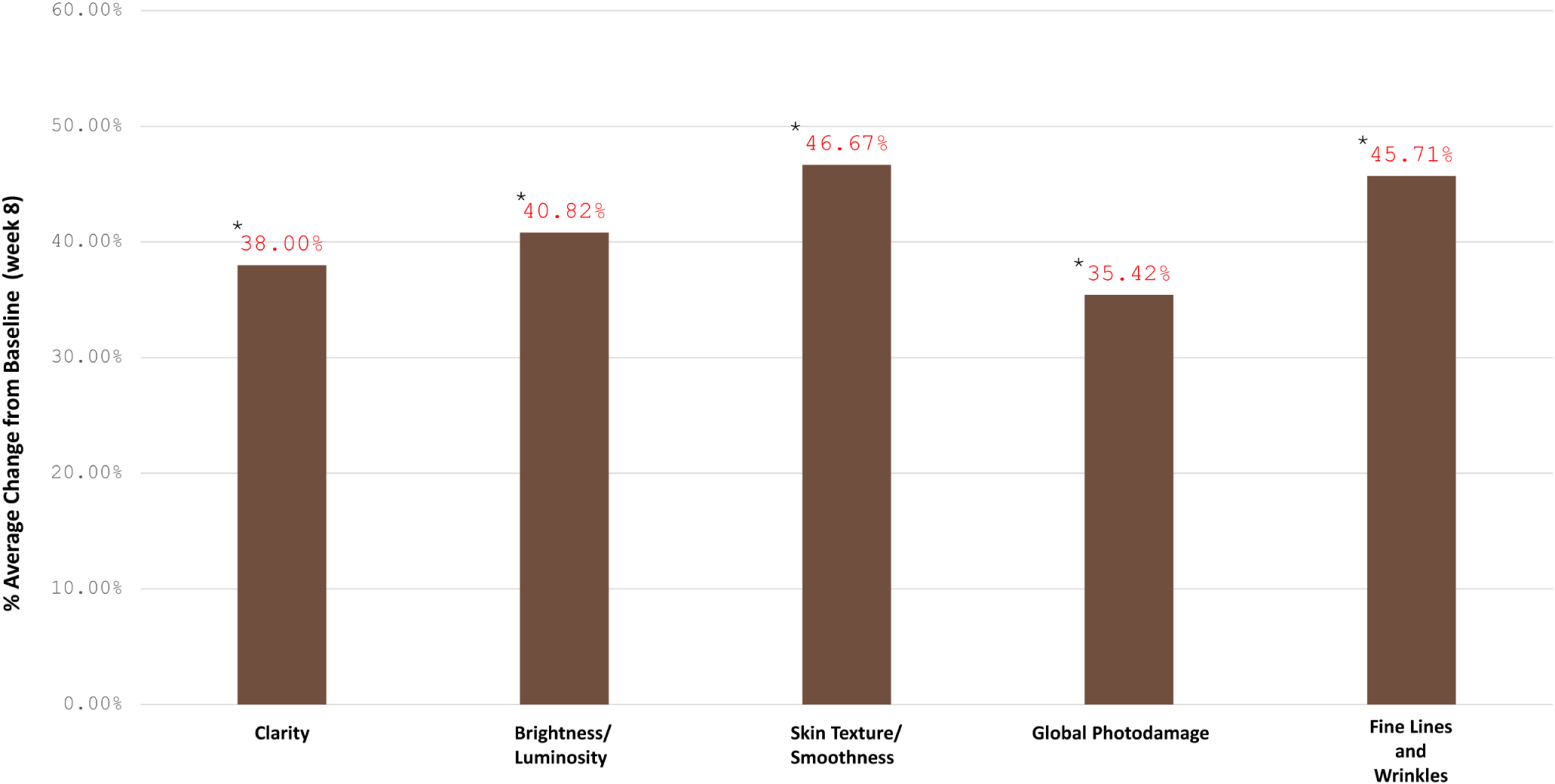
Investigator grading—percentage (%) of average change from baseline. Using a 5‐point scale, clinical investigators graded participants at Visit 4 (Week 8). *Statistically significant (*p* < 0.05) improvement from baseline across all parameters.

**FIGURE 3:**
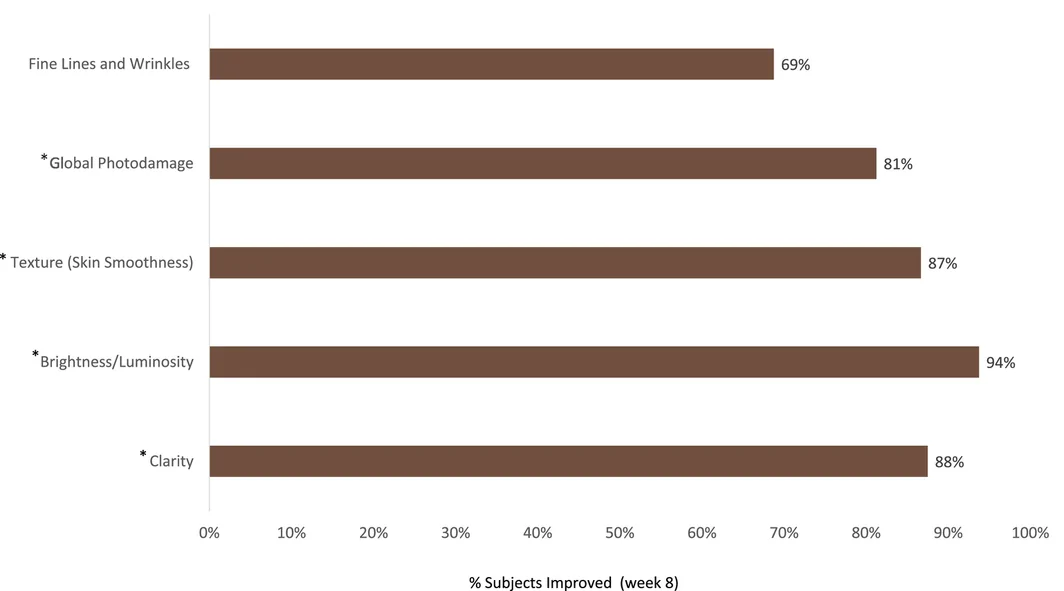
Percentage (%) of subjects improved. The proportion of participants demonstrating improvement remained significant for global photodamage, texture (skin smoothness), brightness/luminosity, and clarity, while fine lines and wrinkles trended toward improvement without maintaining statistical significance. Improvement assessed at Visit 4 (Week 8). *Statistically significant (*p* < 0.05).

**Clinical**: By Week 8 investigator grading demonstrated statistically significant mean improvement from baseline across all five parameters: clarity (+38%), brightness/luminosity (+41%), skin texture/smoothness (+47%), global photodamage (+35%), and fine lines and wrinkles (+46%) (Figure 2). The percentage of subjects that showed improvement was statistically significant for global photodamage (81%), skin texture/smoothness (87%), brightness/luminosity (94%), and clarity (88%) (Figure 3).

**FIGURE 4:**
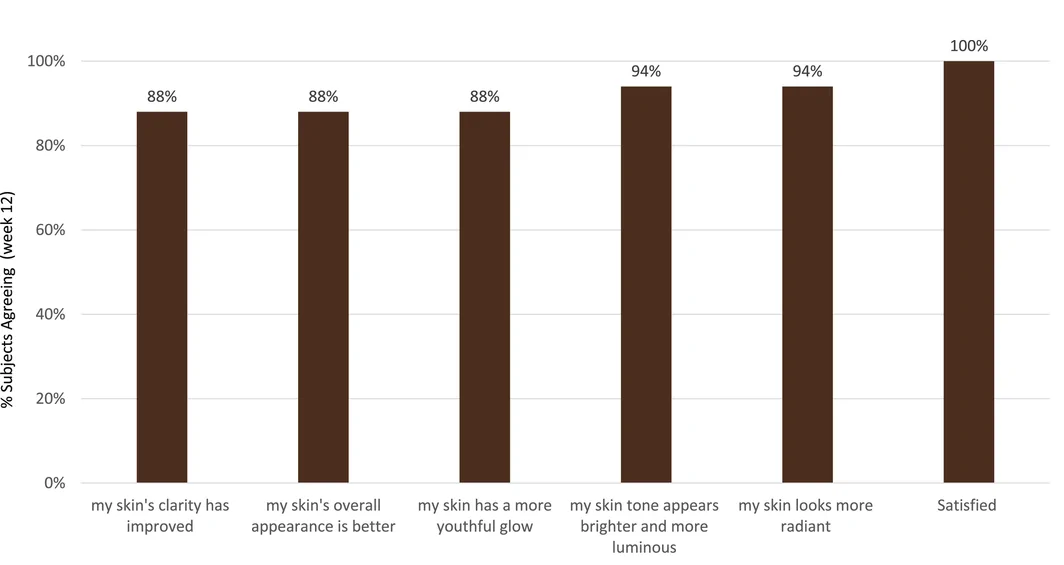
Subject Self‐Assessment Questionnaire responses. Collected at Visit 3 (Week 4), Visit 4 (Week 8), and Visit 5 (Week 12). Representation of Visit 5 (Week 12) responses.

**FIGURE 5:**
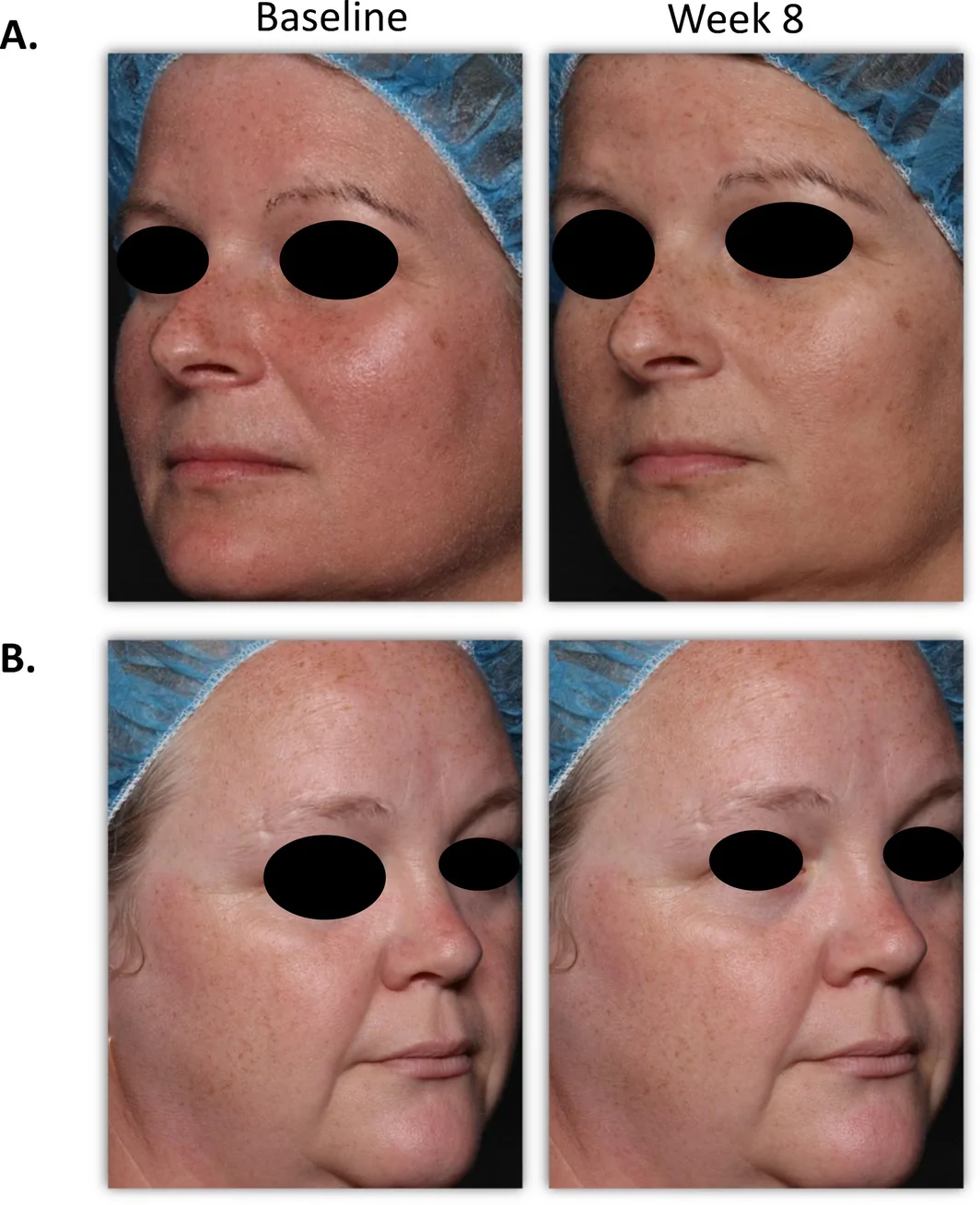
Photographs depicting investigator grading and subject responses at Week 8. Two subjects from Baseline to Visit 4 (Week 8). (A, B) Showing visible improvement in luminosity, clarity, skin texture, tone, and reduced pore size and redness as well as (B) improved clarity. The regimen was well tolerated, with no irritation, erythema, discontinuations, or adverse events observed.

**FIGURE 6:**
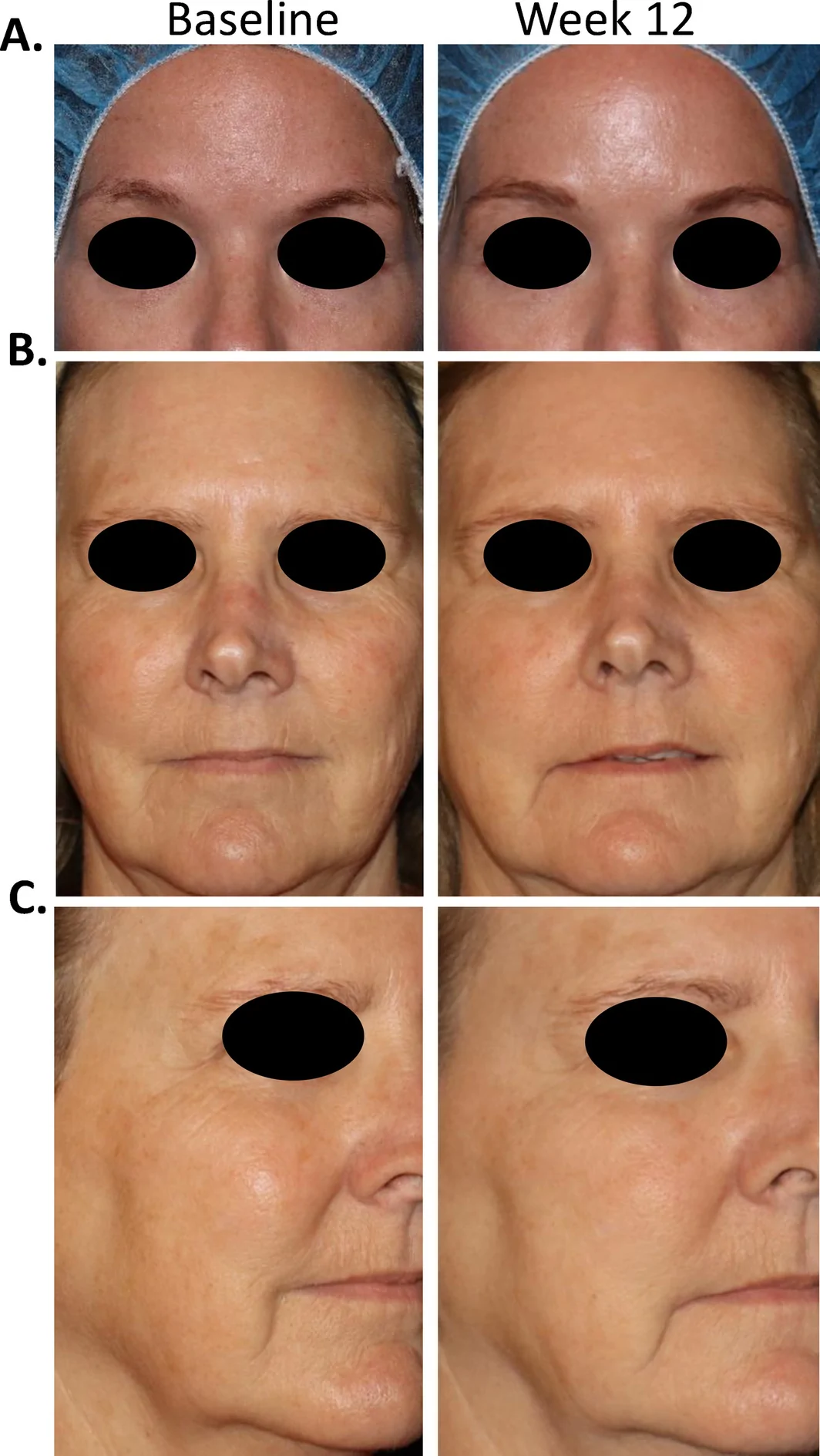
Photographs depicting investigator grading and subject responses at Week 12. Three subjects from Baseline to Visit 5 (Week 12). (A) There was visible reduction in pore size, (A–C) improvement in radiance and skin texture, and (B, C) improvement in global photodamage and wrinkles. The regimen was well tolerated, with no irritation, erythema, discontinuations, or adverse events observed.

At Week 4, 100% of subjects reported no irritation and no skin redness. Subject self‐assessment at the end of the study showed statistically significant agreement: 88% reported improved skin clarity, improved overall appearance, a more youthful glow, while 94% reported increased brightness/luminosity, and radiance (Figure 4). Photographs taken at Week 8 revealed visible improvement in luminosity, clarity, skin texture, tone, as well as reduced pore size and redness (Figure 5A,B) as well as improved clarity (Figure 5B). Additionally, photographs taken at Week 12 showed visible reduction in pore size (Figure 6A), improvement in radiance and skin texture (Figure 6A–C), and improvement in global photodamage and wrinkles (Figure 6B,C). The regimen was well tolerated, with no irritation, erythema, discontinuations, or adverse events observed.

## 2 Discussion

Extrinsic skin aging arises from UV radiation‐induced oxidative stress, DNA damage, and glycation, impairing cellular signaling, extracellular matrix integrity, and redox homeostasis [12]. THD ascorbate, a lipophilic vitamin C derivative, promotes collagen synthesis [10], but is a poor antioxidant that degrades rapidly in oxidative environments [10]. Its inherent instability and activation of type I interferon signaling limit clinical performance [10] and increasing concentrations required to attain true bioactivity can lead to irritation and inflammation in sensitive skin [10]. Passive encapsulation strategies are insufficient to counteract microenvironmental degradation factors [13, 14].

Therefore, single active formulations tend to underperform amid oxidative and inflammatory stressors [15, 16], necessitating a complementary platform to modify the cutaneous microenvironment rather than merely increasing concentrations [4, 9, 10, 17].

Senté's low molecular weight HSA, a mimetic of endogenous heparan sulfate, penetrates epidermis and dermis to recondition the microenvironment: stabilizing growth factor‐receptor interactions, sequestering proinflammatory cytokines, inhibiting inflammasome and NF‐κB pathways, and bolstering extracellular matrix [11, 18].

Beyond its independent biological effects and mechanism of action, HSA enables THD ascorbate to achieve maximal bioactivity by fostering conditions for sustained antioxidant scavenging, collagen hydroxylation, and redox homeostasis. The experimental evidence supports this synergy, demonstrating HSA's capacity to shield THD from oxidative/inflammatory stress, preserving collagen hydroxylation, and averting pro‐oxidant effects to enable higher dosing without cytotoxicity or erythema [10, 16].

Statistically significant reductions relative to UV‐exposed skin (Day 1): 4‐HNE by 44%, gamma‐H2AX by 9.5%, and dermal AGEs by 37% were observed as well as significant clinical improvements from baseline in clarity, brightness, texture, photodamage, and fine lines by Week 8, with 100% participant satisfaction: 88% reporting improvements in skin clarity and glow, 94% noticing increased radiance. No irritation, adverse events, or discontinuations were encountered, thus outperforming THD monotherapy tolerability [10]. However, the study had limitations as it was a modest sample size and it lacked a THD‐only or placebo comparator; larger randomized trials are warranted.

## 3 Conclusion

The co‐formulation of Senté's HSA and THD ascorbate (Defense C Serum) produced statistically significant reductions in UV‐induced oxidative stress, DNA damage, and dermal matrix glycation ex vivo, and clinically meaningful improvements in skin clarity, brightness, and texture over 12 weeks of twice daily use. The combination was exceptionally well tolerated with no adverse events. THD ascorbate and the HSA each independently help reduce the effects of ultraviolet radiation and pollution on skin health through different mechanisms—antioxidant scavenging and signaling modulation. Together, they employ complementary pathways for enhanced protection [19].

These results support the bioactive stacking concept by deliberately pairing mechanistically complementary ingredients to overcome individual limitations. The data herein establishes the foundation for the intentional stacking of complementary and synergistic ingredients with independent mechanisms of action.

## Author Contributions

Conceptualization: V.B., R.G., A.G., A.P.; investigation: V.B., R.G., A.G., A.P.; project administration: V.B., R.G., A.G., A.P.; resources: V.B., R.G., A.G., A.P.; supervision: V.B., R.G., A.G., A.P.; validation: V.B., R.G., A.G., A.P., E.I., A.S.B.; visualization: E.I., A.S.B.; writing – original draft: E.I., A.S.B.; writing – review and editing: V.B., R.G., A.G., A.P., E.I., A.S.B.

## Consent

Informed Consent Form (ICF) was obtained from each participant prior to initiating the study describing reasons for the study, possible adverse effects, associated risks and potential benefits. Signature of a Photography Release Forms were signed, indicating the subject's authorization for release of any photographs obtained during the study.

## Conflicts of Interest

V.B. is an advisory board member for Senté Inc., R.G. is an Investigator for Senté Inc., AbbVie, Galderma, Merz, Aclaris, Symbio, Amgen, Innovaderm, Eli Lilly, Consultant for AbbVie, Cartessa, A.S.B. is a consultant for Senté Inc. All other authors declare no conflicts of interest.

## Data Availability

The data that support the findings of this study are available from the corresponding author upon reasonable request.
